# Translational frontiers: insight from lymphatics in skin regeneration

**DOI:** 10.3389/fphys.2024.1347558

**Published:** 2024-02-29

**Authors:** Yujia Jiang, Mirna Perez-Moreno

**Affiliations:** Section for Cell Biology and Physiology, Department of Biology, University of Copenhagen, Copenhagen, Denmark

**Keywords:** skin, hair follicle, stem cells, regeneration, microenvironment, lymphatics, engineered skin, skin graft

## Abstract

The remarkable regenerative ability of the skin, governed by complex molecular mechanisms, offers profound insights into the skin repair processes and the pathogenesis of various dermatological conditions. This understanding, derived from studies in human skin and various model systems, has not only deepened our knowledge of skin regeneration but also facilitated the development of skin substitutes in clinical practice. Recent research highlights the crucial role of lymphatic vessels in skin regeneration. Traditionally associated with fluid dynamics and immune modulation, these vessels are now recognized for interacting with skin stem cells and coordinating regeneration. This Mini Review provides an overview of recent advancements in basic and translational research related to skin regeneration, focusing on the dynamic interplay between lymphatic vessels and skin biology. Key highlights include the critical role of stem cell-lymphatic vessel crosstalk in orchestrating skin regeneration, emerging translational approaches, and their implications for skin diseases. Additionally, the review identifies research gaps and proposes potential future directions, underscoring the significance of this rapidly evolving research arena.

## Introduction

The skin, a dynamic and multifaceted organ, is widely recognized for its remarkable regenerative capabilities. Its ability to regenerate serves as a foundational knowledge base for stem cell research and is crucial in advancing studies related to wound repair and skin transplantation techniques. Exploring skin regeneration in different model systems and human skin has yielded insights into the complex orchestration of stem cell mechanisms and regenerative processes, shedding light on similar pathways in other tissues. The resilience of skin can be largely attributed to an intricate interplay of biological processes, which encompass immune defense, contraction of fibers and matrix, dynamic fluid transport, and regenerative signals that interact closely with stem cells. This Mini review aims to provide an overview of the recent developments in dermal lymphatics within the context of skin regeneration. It particularly focuses on the newly recognized interactions between lymphatic vessels and hair follicle stem cells. Given the individual significance of hair follicle stem cells and lymphatics in wound repair, this Mini review also outlines potential future research directions, exploring their synergy in wound repair and skin bioengineering. This emerging and evolving field promises to open new avenues in regenerative medicine.

## Skin: a layered complexity

The skin comprises the epidermis, dermis, and hypodermis, serving as a crucial interface between the body’s internal and external environments. Its intricate, multilayered structure endows it with a plethora of functions. As a protective barrier, it shields against pathogenic, mechanical, solar, and toxic threats. Its multilayered structure provides protective, sensory, thermoregulatory, and metabolic functions and immunological defense (Hsu and Fuchs, 2022).

The outermost layer, the epidermis, is primarily composed of epidermal keratinocytes and is interspersed with sweat glands and hair follicles, each exhibiting regenerative capabilities attributable to distinct populations of stem cells (Watt, 2014; Hsu and Fuchs, 2022). The dermis, rich in collagen, elastin fibers, proteoglycans, and hyaluronic acid, provides structural integrity, and harbors fibroblasts, blood and lymphatic vessels, nerves, and immune cells supporting the diversity of skin’s functions (Smith et al., 1982; Skobe and Detmar, 2000; Kupper and Fuhlbrigge, 2004; Prost-Squarcioni et al., 2008; Owens and Lumpkin, 2014; Glatte et al., 2019; Kobayashi et al., 2019; Theocharidis and Connelly, 2019; Plikus et al., 2021; Kam et al., 2023). Situated beneath the dermis, the hypodermis mainly comprises adipocytes, collagen, and blood and lymphatic vessels. It is crucial for structural support, thermoregulation and acts as an energy store, playing endocrine and regenerative roles (Driskell et al., 2014). All layers contribute to skin regeneration and repair, pivotal in research and clinical practice. Lymphatic capillaries and vessels across these layers are essential for fluid and immune transport, with emerging roles in regeneration.

## Skin lymphatic vasculature

The skin possesses a complex network of blood and lymphatic vasculature, each playing distinct yet complementary roles in skin physiology and pathology. The blood vascular system, known for providing oxygen and nutrients and facilitating immune cell traffic, influences the behavior and functionality of epidermal and hair follicle stem cells, impacting wound repair and skin engineering (Tonnesen et al., 2000; Johnson and Wilgus, 2014; Sada et al., 2016; Li et al., 2019; Oualla-Bachiri et al., 2020; Li and Tumbar, 2021; Moreira and Marques, 2022; Wasko et al., 2022; Kam et al., 2023; Li et al., 2023).

**FIGURE 1:**
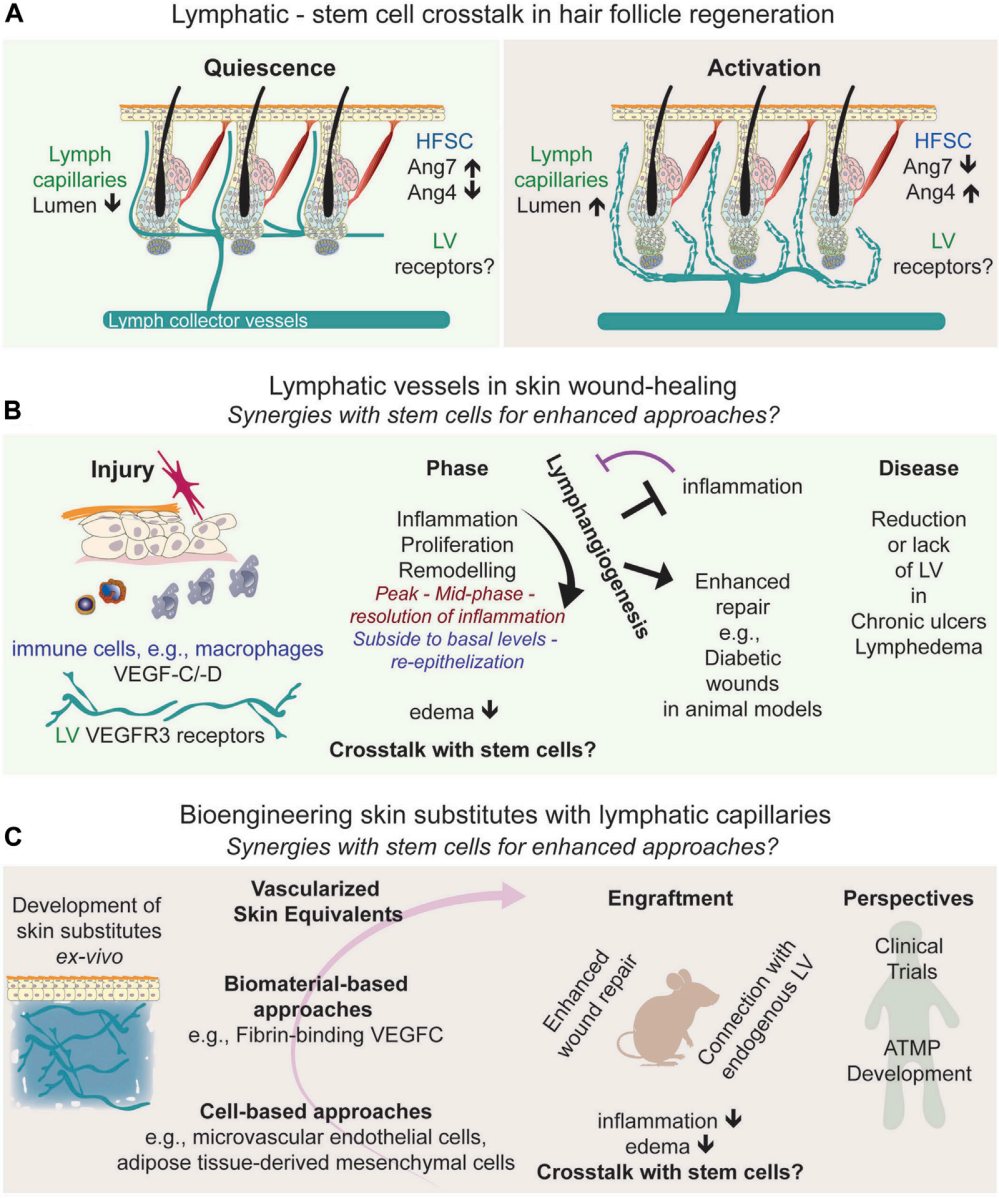
Frontiers in Skin Regeneration: Lymphatic Vessels and Stem Cell Interactions. **(A)** Connections of lymphatic vessels with hair follicle stem cells relevant for skin regeneration, depicting the interactions with bulge stem cells, and the signaling molecules associated to the synergic interactions. **(B)** Lymphangiogenesis in wound healing, **(C)** represents advanced skin grafting techniques, symbolizing the progression into translational approaches, and the integration of lymphatics in regenerative medicine, emphasizing their growing importance in therapeutic approaches and forecasts the future potential of these developments in treating chronic wounds, pointing towards new frontiers in medical research and treatment. Abbreviations: HFSC, Hair follicle stem cell; LV, lymphatic vessels; Ang, angiopoietin; VEGFC/D; Vascular endothelial growth factor C/D; ATMP; Advanced Therapeutic Medical Products.

Dermal lymphatic vessels are organized into two plexuses: a superficial plexus and a deeper subcutaneous plexus. The lymphatic capillary network in the upper dermis, also termed initial afferent lymphatics, connects to larger collecting vessels in the lower dermis and the subcutaneous tissue, facilitating fluid and immune transport (Skobe and Detmar, 2000). The initial afferent lymphatic capillaries also interconnect neighboring hair follicle stem cells across the skin, draining into larger collecting vessels (Figure 1A) (Gur-Cohen et al., 2019; Pena-Jimenez et al., 2019; Yoon et al., 2019). Traditionally viewed as passive conduits for fluid, immune cells, and pathogens, lymphatics also exhibit diverse origins and functions (Oliver et al., 2020; Petrova and Koh, 2020). Recent studies using mouse genetically modified models have highlighted their role in coordinating hair follicle regeneration and growth (Gur-Cohen et al., 2019; Pena-Jimenez et al., 2019; Yoon et al., 2019; Yoon and Detmar, 2022), opening potential avenues for translational research.

## Stem cell and lymphatic vessels crosstalk in hair follicle cycle control

The epidermis, interspersed with sweat glands and hair follicles, contains distinct stem cell populations identified in both mouse and human skin (Lu and Fuchs, 2014; Watt, 2014; Reynolds et al., 2021; Negri and Watt, 2022; Almet et al., 2023; Negri et al., 2023). Hair follicle stem cells, particularly in the bulge region near the sebaceous gland, are well-studied for their regenerative properties, defined in mouse (Cotsarelis et al., 1990; Blanpain et al., 2004; Tumbar et al., 2004) and human skin (Purba et al., 2014; Reynolds et al., 2021; Negri and Watt, 2022; Almet et al., 2023). These multipotent stem cells play a crucial role in the hair follicle cycle, comprising phases of rest (telogen), growth (anagen), and decay (catagen) (Muller-Rover et al., 2001). Studies in mice have revealed that bulge stem cells and progenitor hair germ cells (Greco et al., 2009) have significant regenerative potential, interacting with the dermal papilla (Lei et al., 2017). Their activation initiates the transition from telogen to anagen, leading to the differentiation of transit-amplifying cells into various lineages. Understanding and harnessing the regenerative capacities of bulge stem cells are crucial for promoting endogenous regeneration and advancing stem cell-based therapies. Hair follicle regeneration involves a complex interplay of intrinsic and extrinsic mechanisms (Li and Tumbar, 2021; Hsu and Fuchs, 2022; Zhang and Chen, 2023). The hair follicle’s microenvironment, including its vasculature, significantly influences bulge stem cells (Li and Tumbar, 2021; Yue et al., 2022; Zhang and Chen, 2023). The association of the upper bulge with a perivascular niche is instrumental in sustaining hair follicle stem cells (Xiao et al., 2013), and a population of multipotent stem cells in the bulge can even differentiate into blood vessels (Amoh et al., 2004). However, recent studies have illuminated the role of lymphatic vessels in regulating mouse hair follicle regeneration, expanding their role beyond traditional functions (Gur-Cohen et al., 2019; Pena-Jimenez et al., 2019; Yoon et al., 2019; Yoon and Detmar, 2022). This includes lymphatic vessels responding to Wnt secretion to associate with the stem cell niche and the stem cell secretion of angiopoietin molecules, affecting lymphatic tissue drainage linked to hair follicle regeneration (Figure 1A). Identifying the receptors on lymphatic vessels that lead to changes in tissue drainage will provide further insight into their direct or indirect signaling crosstalk in the skin. The genetic ablation of lymphatic vessels perturbs the hair follicle cycle, potentially due to impairing the concentration gradient of regulatory factors across the skin, and the influx and outflow of immune cells, such as macrophages (Castellana et al., 2014; Wang et al., 2019) and T regulatory cells (Ali et al., 2017; Liu et al., 2022), known to regulate hair follicle regeneration.

Overall, the skin’s microenvironment, heavily influenced by its blood and lymphatic vasculature, is critical in regulating and maintaining hair follicle stem cells. Recent sequencing studies have identified unique factors for lymph vessels compared to blood vessels (Chovatiya et al., 2023). This emerging knowledge holds the potential for exploring the distinct roles of lymphatics-stem cell crosstalk in skin regeneration and their implications in dermatological diseases, including skin cancer (Cazzola et al., 2023) and aging (Kataru et al., 2022), opening new avenues for potential translational studies in skin regeneration.

## Blood vasculature and epidermal stem cells: paving the way for understanding lymphatic involvement?

In the stratified epidermis, maintaining the balance between cell proliferation and terminal differentiation is crucial for the continuous renewal of skin cells and enhancing regenerative capabilities. Basal progenitor cells can undergo symmetric divisions to preserve the skin surface area or differentiate into the suprabasal spinous, granular, and corneal layers (Jones et al., 2007; Blanpain and Fuchs, 2009; Simpson et al., 2011; Gandarillas et al., 2018; Cockburn et al., 2022; Prado-Mantilla and Lechler, 2023).

Interestingly, the blood vasculature influences epidermal stem cell behavior, while the role of lymphathic vessels remains poorly understood. Some insights gained from the blood vasculature regarding sustaining skin homeostasis could potentially pave the way for exploring the complementary roles of lymphatic vessels in epidermal stem cell proliferation.

In mouse skin, two distinct basal progenitor stem cell territories exist (Mascre et al., 2012; Gomez et al., 2013; Roy et al., 2016; Sada et al., 2016), with conserved expression patterns with human skin (Ghuwalewala et al., 2022). The mouse inter-scales, equivalent to human inter-ridges, express the Sox6 gene, forming UV-responsive proliferating clusters. Conversely, mouse scales, with non-label retaining properties, equivalent to human rete ridges are enriched with specific gene signatures like Slc1a3, showing resilience to UV stress (Ghuwalewala et al., 2022). Interestingly, mouse scales, analogous to human rete ridges, are located near dense blood vessel arrays, suggesting significant stem cell-blood vessel interactions (Sada et al., 2016).

Mechanical stretch has been shown to induce skin stem cell renewal (Aragona et al., 2020), and blood vessels in abdominal skin promote the formation of epidermal proliferating clusters, which are enhanced by mechanical stretch (Ichijo et al., 2021). In contrast, blood vessel atrophy during aging leads to dermal stiffening and differentiation of basal progenitor stem cells (Ichijo et al., 2022).

These findings underscore the coordinated interaction between epidermal stem cells and blood vasculature in maintaining skin homeostasis and responding to stresses like UV exposure and mechanical stretch.

The role of lymphatic vessels in these processes remains unexplored. The distinct LV territorial distribution in humans and other species (Suami et al., 2007; Soto-Miranda et al., 2013; Suami et al., 2013; Ito and Suami, 2015; Suami, 2017; Suami and Scaglioni, 2017) raises the plausible speculation that lymphatic vessels, like their blood vessel counterparts, may exert similar influences on different basal progenitor cell territories and play a significant role in epidermal regeneration. Such a hypothesis presents a compelling direction for future research to explore the influence of local microenvironments (Lawlor and Kaur, 2015; Roy et al., 2016; Cheng et al., 2018) and different stem cell population responses to mechanical and environmental stress, including mechanical stretch and wound healing (Aragona et al., 2017; Park et al., 2017; Aragona et al., 2020; Gola and Fuchs, 2021; Ghuwalewala et al., 2022).

## Lymphatic vessels in skin wound healing: new avenues to explore the synergies with stem cells?

In the multifaceted process of skin wound healing, the re-epithelialized wound epidermis consists of progeny from both epidermal and hair follicle stem cells, as well as de-differentiated cells (Blanpain et al., 2004; Tumbar et al., 2004; Donati et al., 2017; Sun et al., 2023) that repopulate the tissue to achieve wound repair.

During wound healing, lymphangiogenesis, the formation of new lymphatic vessels, is also vital for restoring skin and vascular functionality. Lymphatic vessels are crucial in reducing interstitial pressure and edema by facilitating fluid drainage. They also play a significant role in regulating immune cell transport and responses, positioning them as key therapeutic targets in skin regeneration (Reno and Sabbatini, 2023). Investigating the potential functional relationship between lymphatic vessels and skin stem cells during wound healing could yield further insights into lymphatic-mediated wound repair mechanisms. It is established that lymphangiogenesis and angiogenesis concurrently increase during wound repair (Tonnesen et al., 2000; Cho et al., 2006; Johnson and Wilgus, 2014; Wasko et al., 2022). In the case of lymphangiogenesis, wound-induced inflammation or infections trigger cytokine secretion and the recruitment of innate immune cells, and the secretion of cytokines and the lymphangiogenic factors VEGF-C/-D that bind to VEGF-R3 receptors on lymphatic endothelial cells (Paavonen et al., 2000; Kataru et al., 2009) (Figure 1B). Lymphangiogenesis peaks in the mid-phase to the resolution of inflammation and subsides to basal levels upon completion of re-epithelization (Martinez-Corral et al., 2012) (Figure 1B). Yet, the specific contributions of lymphatic vessels and their molecular signals in each phase of wound healing remain to be fully elucidated.

Recent findings highlight a strong correlation between the inflammatory response and lymphangiogenesis. Anti-inflammatory treatments block lymphangiogenesis (Martinez-Corral et al., 2012) while promoting lymphangiogenesis counteracts inflammation (Huggenberger et al., 2010; Huggenberger et al., 2011). Additionally, the loss of lymphatic vessels can induce a proinflammatory environment, delaying wound closure (Brunner et al., 2023). This suggests a dynamic and reciprocal relationship between lymphatic function and the inflammatory process in wound healing (Figure 1B).

Examining the intricacies of lymphatic crosstalk with other microenvironmental cells, such as macrophages, could be particularly revealing to provide insight into the complex interactions in skin repair connecting stem cells. Macrophages are crucial in wound-induced *de novo* hair follicle stem cell regeneration (Chen et al., 2015; Wang et al., 2017; Rahmani et al., 2018) and are key regulators of lymphangiogenesis during wound healing (Paavonen et al., 2000; Kataru et al., 2009; Wiig et al., 2013; Hadrian et al., 2021). Understanding the spatio-temporal dynamics between these populations could deepen our knowledge in the context of wound-induced hair regeneration and offer translational implications.

While much of the research mentioned above is based on studies with mice and genetically modified mouse models, these have been crucial in uncovering the role of lymphatic vessels in wound healing. This research opens new possibilities for treating chronic wounds, where normal healing processes are often disrupted. In human skin, chronic ulcers lack lymphatic vessels (Brunner et al., 2023). Mathematical modeling of lymphangiogenesis in diabetic human wounds suggests potential applications for treating such conditions (Bianchi et al., 2015). Additionally, single-cell transcriptomic analyses of human diabetic foot wounds have revealed significant alterations in various signaling pathways, including those related to lymphatic vessels (Sandoval-Schaefer et al., 2023), underscoring the importance of exploring the mechanisms regulating lymphangiogenesis for the treatment of diabetic wounds. In mouse models, a clear link has been established between impaired diabetic wound healing and hindered lymphatic vessel formation due to reduced macrophage activity (Maruyama et al., 2007). Conversely, diabetic wound healing improves when lymphangiogenesis is stimulated (Figure 1B), either through pharmacological means or by using lymphangiogenic factors (Reno and Sabbatini, 2023), such as COMP-Angiopoietin1 (Cho et al., 2006) and VEGF-C (Saaristo et al., 2006; Guc et al., 2017; Lim et al., 2019; Brunner et al., 2023).

In conclusion, the role of lymphatic vessels in skin wound healing is multifaceted and crucial. They are involved in fluid regulation, waste removal, and the modulation of immune responses. While our understanding of their functions in human wound healing is still developing, investigating the spatiotemporal roles of lymphatics in various stages of wound repair, along with their interactions with bulge stem cells, represents a promising and complex area in regenerative medicine to enhance wound repair. This is particularly relevant for chronic wounds and vascular diseases like lymphedema. As research progresses, these insights have the potential to lead to innovative therapeutic strategies, enhancing wound healing and tissue regeneration.

## Merging skin grafts and bioengineering perspectives with lymphatic insights: facilitating synergies with stem cells for enhanced approaches?

Skin transplantation and bioengineering approaches have evolved significantly, transitioning from traditional grafting techniques to sophisticated bioengineering methods for developing skin substitutes to treat extensive skin defects. This shift, which began with the clinical use of lab-grown keratinocytes in the 1980s (Watt, 2014), has led to remarkable progress in cellular and tissue-engineered therapies for skin regeneration (De Rosa and De Luca, 2022).

The integration of advanced materials and technologies, including *in situ* 3D printing, portable bioprinters, and electrosprayers, into cellular therapies is revolutionizing wound healing and skin regeneration (Chouhan et al., 2019). Despite these advancements, the challenge remains to create a skin substitute that fully replicates all the biological characteristics of native skin.

In light of the lymphatic insights discussed earlier, it is evident that fostering synergies with stem cells represents a promising avenue for enhancing approaches in skin grafts and bioengineering perspectives. Recent tissue engineering strategies are increasingly focusing on vascularized skin equivalents, and efforts to promote neotissue vascularization during tissue regeneration are employing innovative biomaterial-based and cell-based approaches (Oualla-Bachiri et al., 2020; Moreira and Marques, 2022).

Due to the significant roles of lymphatic vasculature in skin repairs, their potential to enhance the success of skin grafts and dermal substitutes has recently garnered essential interest (Figure 1C). Engineered fibrin-binding VEGF-C, for instance, has been shown to promote wound healing by increasing immune cell trafficking and matrix remodeling (Guc et al., 2017). A pivotal advancement in skin bioengineering was incorporating microvascular endothelial cells, encompassing both blood and lymphatic endothelial cells, into grafts. This innovative approach created dermo-epidermal skin grafts with integrated lymphatic vessels, which successfully vascularized *ex vivo*. When transplanted onto wounds in nude rats, these grafts connected with existing lymphatic capillaries, significantly enhancing fluid drainage (Marino et al., 2014). This breakthrough represents a major advancement in developing grafts that closely replicate the natural functionality of the skin. The technique led to the creation of full-thickness skin analogs that mimic the physiological, structural, and functional properties of native skin. These developments address challenges like rapid graft acceptance and effective nutrient and oxygen delivery through pre-established capillary networks.

Furthermore, additional approaches involving adipose tissue-derived isolates has been shown to enhance lymphatic drainage and immune cell trafficking in dermal substitutes. This method stimulates the integration of implants onto mouse wounds, thereby enhancing microvascular network formation and skin regeneration (Frueh et al., 2017; Asano et al., 2023).

## Discussion

The facilitation of lymphangiogenesis in skin wound healing presents a multifaceted approach to medical intervention, particularly in reducing edema and promoting debris removal. However, the broader implications of lymphatic vessels, especially their contribution to stem cell activation during the repair process, warrant further exploration. This exploration becomes even more compelling when considering the dynamic interaction of lymphatic vessels with immune cells in the transplanted wound microenvironment. Macrophages, for instance, could significantly enhance the integration process of transplanted tissues, thereby improving the outcomes of skin transplantation and wound repair.

The integration of fundamental knowledge from skin cell and molecular biology, combined with insights into stem cell and lymphatic vessel physiology, is notably advancing the field of regenerative medicine. This synergy is expanding the scope of therapeutic possibilities and offering novel solutions for chronic wounds and skin diseases. Looking ahead, the potential of lab-made grafts and Advanced Therapeutic Medical Products, currently in clinical trials, could be further augmented by incorporating lymphatic vessels. This strategic inclusion aims to replicate the native skin’s functionality more accurately and could improve the efficacy of skin transplantation and regenerative therapies.

As this field evolves, a deeper understanding of the interplay between lymphatic networks, stem cells, and immune responses in wound healing will be crucial. Such knowledge could pave the way for innovative treatments tailored to individual healing processes and specific wound types, especially in chronic wounds where traditional approaches have limited efficacy.
